# Neuro-humoral control during orthostasis in health and disease

**DOI:** 10.3389/fphys.2014.00521

**Published:** 2015-01-12

**Authors:** Qi Fu

**Affiliations:** ^1^Institute for Exercise and Environmental Medicine, Texas Health Presbyterian Hospital DallasDallas, TX, USA; ^2^Cardiology Division, Internal Medicine, University of Texas Southwestern Medical CenterDallas, TX, USA

**Keywords:** sympathetic neural activity, renal-adrenal, blood pressure, syncope, orthostatic intolerance, orthostatic hypotension

Different from many other species, human beings spend nearly two-thirds of the day in an upright posture. Due to gravity, about 700 to 900 ml of blood shift into the lower body during quiet standing. As a result, central blood volume decreases and this leads to a series of neural-humoral adjustments that aim to maintain the mean arterial pressure during orthostasis. Both the sympathetic nervous system and the renin-angiotensin-aldosterone system contribute importantly to the arterial pressure maintenance in upright humans. There is a growing interest in the blood pressure regulation during orthostasis in healthy individuals and patient populations. This e-book provides some updates on sympathetic neural control and renal-adrenal function in health and disease.

Sympathetic neural activity regulates blood pressure mainly through the baroreflex-mediated vasoconstriction during orthostasis. The baroreflex is a closed-loop feedback system, but requires open-loop experiments to identify its characteristics, which is impossible in human research. Kamiya et al. demonstrated that system identification based analysis and numerical simulation using baroreflex subsystem characteristics identified in animal models may contribute to our understanding of human sympathetic physiology under orthostasis (Kamiya et al., 2014).

Neural and renal-adrenal control of blood pressure can be affected by not only age but also race and physical fitness. For example, it was found that young normotensive Caucasian women may rely on sympathetic neural activity more so than African-American women who had a tendency to rely on the renal-adrenal system to regulate blood pressure during orthostatic challenges (Jarvis et al., 2014). Conversely, Sugawara et al. reported that endurance-trained men had increased orthostatic tachycardia and enhanced cardiovagal baroreflex sensitivity, which may favorably mitigate accumulated risks for post-exercise orthostatic intolerance (Sugawara et al., 2014).

Failure of the sympathetic nervous system during orthostasis may result in syncope (Iwase et al., 2014). However, orthostatic tolerance does not seem to be related to resting sympathetic neural activity or sympathetic baroreflex sensitivity (Hinojosa-Laborde et al., 2014). Studies in healthy young individuals indicate that in addition to sympathetic vasoconstriction, the heart rate response may also contribute importantly to orthostatic tolerance (Convertino, 2014). On the other hand, it is argued that cardiac effector mechanisms appear not to be important for the adjustment of arterial pressure to the upright posture in humans (Wieling et al., 2014). Indeed, investigations into the role of the sympathetic nervous system in orthostatic intolerance have yielded mixed results. The review article by Lambert and Lambert (2014) outlines the current knowledge of the function of the sympathetic nervous system in vasovagal syncope and the Postural Orthostatic Tachycardia Syndrome (POTS, also called Chronic Orthostatic Intolerance).

There are multiple pathophysiological mechanisms that underlie POTS; some POTS patients have evidence of elevated sympathoneural tone, others have a hypovolemic state, and many patients have a perturbed renin-angiotensin-aldosterone system profile (Mar and Raj, 2014). Patients with POTS have acute changes in cognitive performance in response to orthostatic challenges, and these patients also have an increased prevalence of depression and higher levels of anxiety (Anderson et al., 2014). Impaired cerebral autoregulation may underlie upright cognitive dysfunction in POTS (Medow et al., 2014).

Finally, patients with autonomic failure often have orthostatic hypotension and (pre)syncopal symptoms. Arnold et al. showed that ergotamine combined with caffeine could be used as an alternate treatment in carefully selected patients without comorbid coronary artery disease (Arnold et al., 2014).

Taken together, this e-book highlights only a small portion of the recent advances in neural-humoral control during orthostasis in humans. More research is needed to better understand the role of the sympathetic nervous system and the renin-angiotensin-aldosterone system in the regulation of blood pressure in health and disease.

## Conflict of interest statement

The author declares that the research was conducted in the absence of any commercial or financial relationships that could be construed as a potential conflict of interest.
